# Dynamic accommodation measurement using Purkinje reflections and machine learning

**DOI:** 10.1038/s41598-023-47572-0

**Published:** 2023-12-07

**Authors:** Faik Ozan Ozhan, Ugur Aygun, Afsun Sahin, Hakan Urey

**Affiliations:** 1 Department of Electrical and Electronics Engineering, Koç University, 34450 Istanbul, Turkey; 2 Koç University Translational Medicine Research Center (KUTTAM), 34450 Istanbul, Turkey; 3 School of Medicine, Koç University, 34450 Istanbul, Turkey

**Keywords:** Biophotonics, Translational research, Object vision

## Abstract

Quantifying eye movement is important for diagnosing various neurological and ocular diseases as well as AR/VR displays. We developed a simple setup for real-time dynamic gaze tracking and accommodation measurements based on Purkinje reflections, which are the reflections from front and back surfaces of the cornea and the eye lens. We used an accurate eye model in ZEMAX to simulate the Purkinje reflection positions at different focus distances of the eye, which matched the experimental data. A neural network was trained to simultaneously predict vergence and accommodation using data collected from 9 subjects. We demonstrated that the use of Purkinje reflection coordinates in machine learning resulted in precise estimation. The proposed system accurately predicted the accommodation with an accuracy better than 0.22 *D* using subject’s own data and 0.40 *D* using other subjects’ data with two-point calibration in tests performed with 9 subjects in our setup.

## Introduction

Eye movements are essential for our visual system to function correctly. Eyes converge to fixate on points in space during these movements. The distance between a specified point and the eyes is called the vergence distance, and the amount of rotation during this process is known as the vergence angle. The quantification of eye movements and vergence is essential in the diagnosis and management of many neurological diseases such as multiple sclerosis^1,2^, Parkinson’s disease^3,4^, ocular diseases such as strabismus^5,6^, nystagmus^7–9^, and visual impairments such as amblyopia^5,10,11^ or achromatopsia^12^. Eye movements are coupled with the focusing mechanism of the eye. The effective focal length of the eyes is adjusted by a change in the shape of the human crystalline lens, which is controlled by the ciliary muscles. This process is called accommodation, and the distance at which the eyes are focused is called accommodation depth measured in diopters (*D*), which is the reciprocal of the focus distance in meters. Accommodation depth typically varies between 4 *D* (25 cm) and 0 *D* (infinity) in adults. Accurately measuring the accommodation of the eye is crucial for understanding refractive errors^13–15^. Therefore, objectively measuring vergence and accommodation responses is beneficial in both research and clinics.

The accommodation and vergence are coupled and follow one another. Vergence can be predicted using eye movements and gaze angle. However, discrepancies are present between accommodation and vergence in certain situations, such as with 3D displays. In the case of 3D displays, the viewers’ accommodation distance is at a 3D screen in contrast to the vergence distance, which is at the object’s apparent distance in 3D. This phenomenon is referred to as vergence-accommodation conflict (VAC)^16^, and its effects on various tasks have been analyzed in different studies^17–20^. Therefore, while vergence can be predicted with high accuracy using eye movements, accommodation cannot always be predicted accurately. Consequently, an alternative method is required for precise accommodation measurement.

Autorefractors are a commonly used technique for measuring accommodation by examining how light is refracted by the eye to determine refractive errors^21–23^. Although widely used in clinical assessments, autorefractors rely on retinoscopy, a method involving the projection of a light beam into the eye and observing the light reflected from the retina. As a result, they do not provide information about the shape and optical power of the human crystalline lens during the accommodation process without a reference from the unaccommodated state. Moreover, many clinical autorefractors are bulky, limiting continuous monitoring and evaluation of accommodation during different activities and lacking information about vergence.

**Figure 1:**
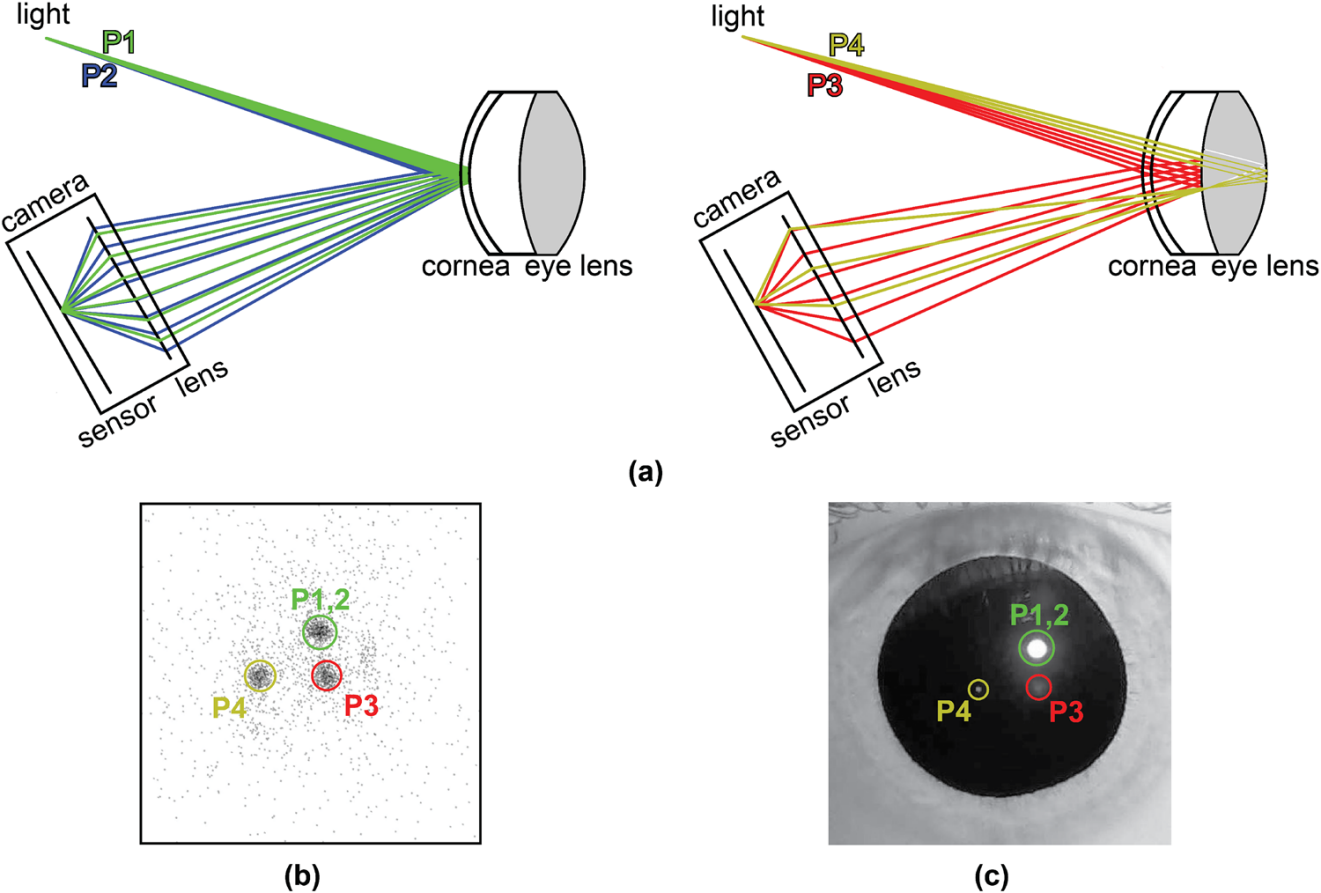
(**a**) Ray tracing simulation layouts illustrating 4 Purkinje reflections. (**b**) Detector view using the eye model in (**a**). (**c**) Experimental result illustrating Purkinje image formations using NIR illumination.

Accommodation depth and vergence angle can be measured using the Purkinje reflections from the cornea and the eye lens illuminated by a point light source and measured using a camera focused on the pupil. Specifically, the first Purkinje image (P1) is the reflection from the anterior, the second Purkinje image (P2) from the posterior surface of the cornea, the third Purkinje image (P3) from the anterior, and the fourth Purkinje image (P4) from the posterior surface of the human crystalline lens. It should be noted that P1 and P2 are indistinguishable since they are formed very closely to each other, and the combination of them forms the brightest spot. ZEMAX simulations are performed to simulate these reflections, and one NIR (near-infrared) LED source and one NIR camera are used to capture Purkinje images experimentally. Figure 1 shows ZEMAX layouts demonstrating how Purkinje reflections are formed on the camera, the image formed on the camera sensor (from ZEMAX detector view), and the experimental result illustrating Purkinje image formations using NIR illumination.

The locations of Purkinje images are highly related to the shape of the human crystalline lens. As the eye accommodation depth changes, the shape of the human crystalline lens changes. More specifically, as the subject focuses on near distances, the ciliary muscles contract, and the curvature of the lens increases. Researchers have developed accommodation-dependent models of the human eye by measuring the corneal radius of curvature, lens radii of curvature, and lens thickness as a function of accommodation^24–26^. Additionally, rotational movement of the eye occurs when the subject looks at different points, resulting in changes in the locations of reflections. Since P3 and P4 are formed after some refraction processes, the locations of P3 and P4 change significantly, while P1 and P2 remain almost stationary with eye rotation.

Prior work has shown that Purkinje reflections can be used for vergence estimation and eye-tracking^27,28^. With the rise of machine learning (ML) and deep learning techniques, researchers have explored the use of these methods on Purkinje reflections to develop an eye tracker capable of predicting accommodation depth as well^29–31^. However, these studies use either P1 with P3^29,30^ or P1 with P4^27,28,31^ while discarding the other reflection. As a result, data and the accuracy of model predictions have been limited. In our previous study, we proposed a primitive version of our work, illustrating that accommodation can be measured dynamically using Purkinje images for different subjects^32^. However, the accommodation depth predicted with the previous ML model, trained with the subject’s own data, and resulted in higher error than the current version of our work, and vergence was only predicted with the feature extracted from P3 and P4 for one subject, not using ML.

In this work, we present a novel dynamic eye accommodation depth and vergence measurement system based on Purkinje reflections and the ML model. In order to achieve user-independent dynamic accommodation measurement, we propose a simple optical setup consisting of a NIR LED illuminating the eye, a camera to capture Purkinje reflections, and a controllable RGB light source array with target points. We used the pupil center and the first 2 Purkinje reflections (P1 and P2) from the cornea and higher-order reflections (P3 and P4) from the eye lens for our measurements. In addition to AR/VR applications, the proposed system can be used in vision science applications and in multifocal intraocular lens simulators for cataract patients^33^ for dynamic accommodation and vergence measurement.

## Results

### The relationship between Purkinje images and accommodation/vergence

We designed a setup that enables us to relate Purkinje images to both accommodation and vergence. Details of the optical setup are given in the Methods section. To simulate the changes in Purkinje reflections with accommodation, we modified Navarro’s accommodation-dependent eye model^24^. The Navarro model consists of four refracting surfaces, making it convenient to simulate Purkinje reflections. In addition, the model parameters are well-established and accommodation-dependent, enabling continuous observation of changes with accommodation. However, the ocular media in the model are designed for visible wavelengths. The refractive indices of the ocular media at NIR were adjusted to make the Purkinje reflection locations more accurate compared to our experiments. The axial length from the cornea to the retina was kept constant. In contrast, other model parameters (radius of curvature of the eye lens, lens thickness, anterior chamber length, and refractive indices of the ocular media) were changed to make focus adjustments (see Supplementary Table S1 online). It should be noted that these parameters can vary between subjects due to anatomical variations in the eye. Eye rotation was also modeled. If the distance to a target point is *d* and the distance between the optical axis of the eye and the target point is $d_{target}$, the rotation angle is calculated using Eq. (1) to take vergence into account in our model. We assumed that the height of the target point was properly adjusted with respect to the eyes, so the rotation only occurred in one direction.

1 $$\begin{aligned} \theta = arctan\left( \frac{d_{target}}{d}\right) \end{aligned}$$

**Figure 2:**
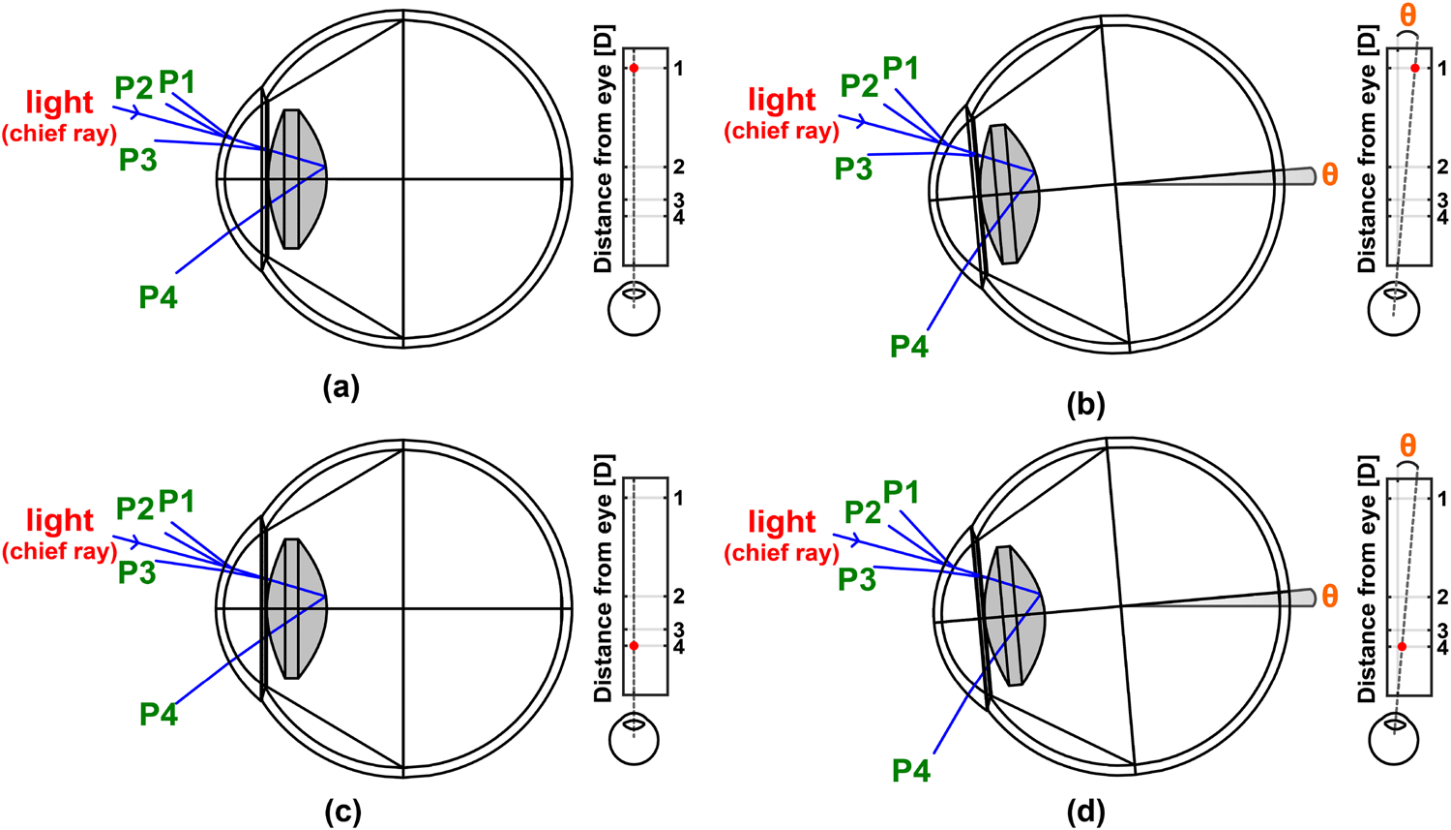
Simulation layouts and optical paths illustrating chief ray reflections from 4 different optical layers for different accommodation and vergence conditions. (**a**) Focus of the eye at 1D (distance vision) with no eye rotation. (**b**) Focus of the eye at 1D (distance vision) with vergence angle of 5 deg. (**c**) Focus of the eye at 4D (near vision) with no eye rotation. (**d**) Focus of the eye at 4D (near vision) with vergence angle of 5 deg.

Figure 2 illustrates the directions of Purkinje reflections for different accommodation and vergence values, as predicted by our eye model, corresponding to the target point depicted by the small inset to the right of each subfigure. The optical path in each inset illustrates the top view of the eye and the target point. Note that the most significant change due to accommodation occurs in the reflection from the anterior lens surface (P3). Importantly, the relative positions of the eye, camera, and light source can affect the specific changes in the Purkinje image locations, and different setups would yield different results. We configured the position of the camera and the light source to achieve a large angular separation without causing vignetting for P3 and P4 reflections, especially when the pupil size is small.

**Figure 3:**
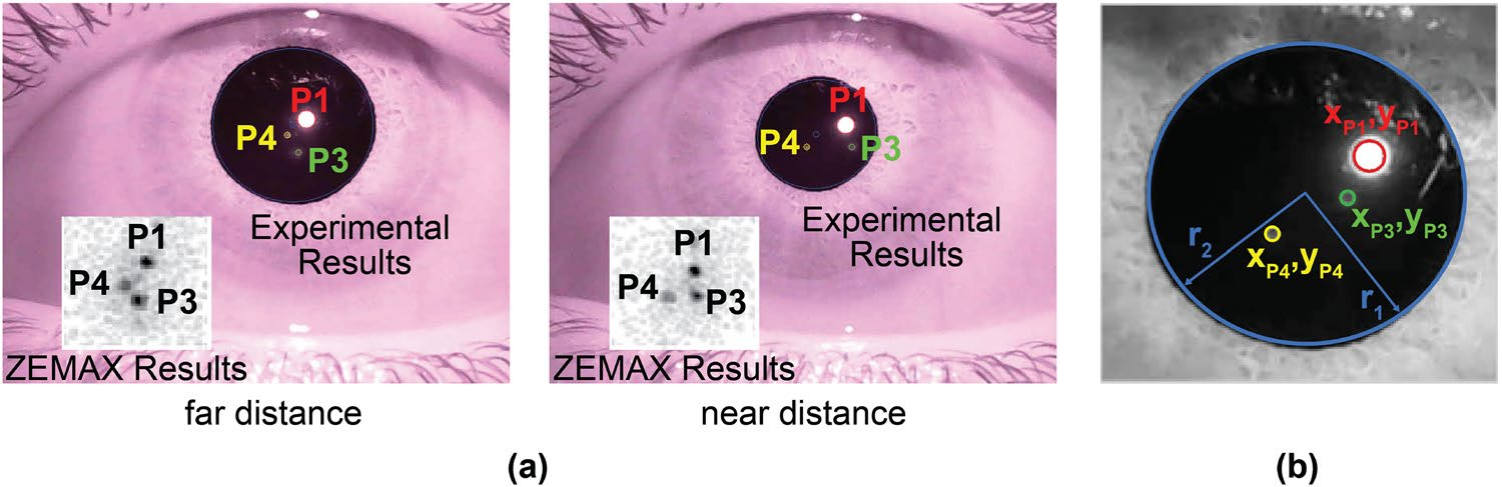
(**a**) Image processing algorithm results and ZEMAX eye model predictions (figure inset) are compared and they are in good agreement . (**b**) Coordinates of 4 points (pupil center, P1, P3, and P4) extracted with our algorithm, which are used as input to the machine learning algorithm.

The captured images on the camera were processed using image processing techniques to identify Purkinje reflections. ZEMAX non-sequential ray tracing simulations resulted in similar changes in Purkinje reflection locations in response to changes in accommodation and vergence, as illustrated in Fig. 3a. Based on these results, we identified 10 parameters, which are pupil center and size $(x_{PC}, y_{PC}, r_{1}, r_{2})$ and locations of Purkinje images $(x_{P1}, y_{P1}, x_{P3}, y_{P3}, x_{P4}, y_{P4}).$ The use of 2 parameters $r_{1}, r_{2}$ for pupil size may seem confusing at first; however, it is necessary to accurately describe the size and the elliptical image of the pupil in our off-axis imaging system. These parameters and their dependence on accommodation and vergence changes can be seen in Fig. 3b.

### Accommodation and vergence prediction using features extracted from the Purkinje images

**Figure 4:**
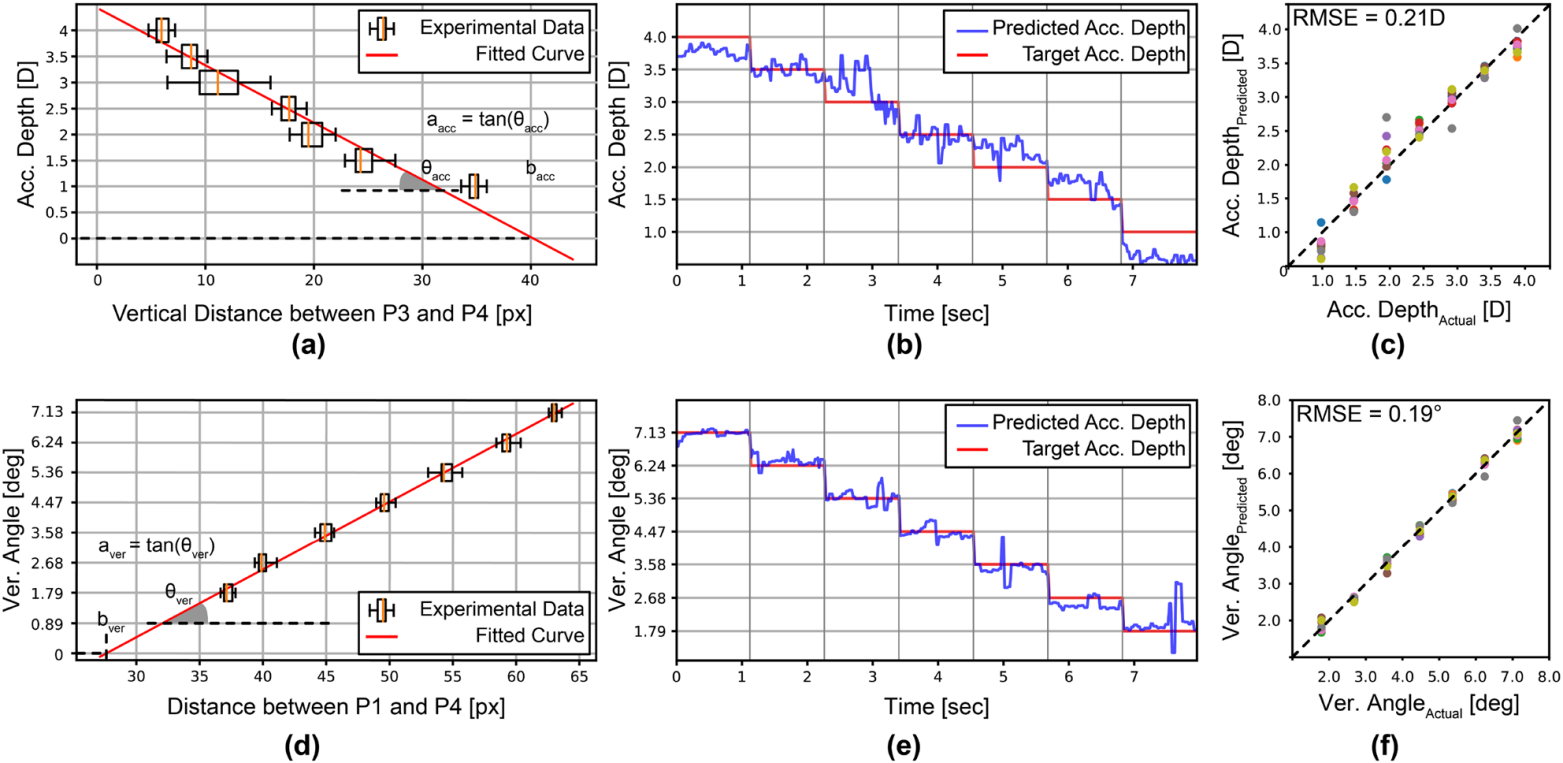
Accommodation and vergence predictions using analytical formulas (without machine learning) using data for 9 subjects (**a**) The vertical distance between P3 and P4 ($y_{P3}-y_{P4}$) obtained from the experimental results of one subject is correlated with accommodation distance of the target LED in the setup. (**b**) Predicted accommodation depth as a function of time using ($y_{P3}-y_{P4}$) (red line shows target LED distance) for one subject. (**c**) Mean values of predicted accommodation depths at all target accommodation depth levels for all 9 subjects. (**d**) The Euclidean distance between P1 and P4 ($d(\textrm{P3}-\textrm{P4})$) obtained from the experimental results of one subject is correlated with vergence angle of the target LED. (**e**) Predicted vergence angle as a function of time using ($d(\textrm{P3}-\textrm{P4})$) (red line shows target LED distance). (**f**) Mean values of predicted vergence angles at all target vergence angle levels for all 9 subjects.

Before applying the ML algorithms, we first expressed the accommodation and vergence angles as functions of the features extracted from the parameters detailed in the previous section. To identify which features were most correlated with accommodation and vergence, we conducted correlation analyses between the different extracted features and the target accommodation depths for the left eyes of 9 subjects. This allowed us to obtain a matrix of correlation coefficients, which revealed that the vertical distance between P3 and P4 in our configuration was highly correlated with accommodation, while the distance between P1 and P4 was highly correlated with vergence. The unit of measure for distances is pixels (px). Using these findings, we calculated these features for all the experimental results and were able to express the linear relationship between these features and accommodation/vergence for each subject individually. Figure 4a,d illustrates the extracted features from experimental results with corresponding target accommodation/vergence values and curves based on linear relationships for one of the subjects. Mathematically, the resulting function for accommodation prediction is expressed in Eq. (2) where $a_{acc}$ (*D*/pixels) represents the slope of the curve, $b_{acc}$ (pixels) the x-intercept, as illustrated in Fig. 4a. The resulting function for vergence prediction is expressed in Eq. (3), where $a_{ver}$ (deg/pixels) is the slope of the curve corresponding to Eq. (3), and $b_{ver}$ (pixels) is the x-intercept, as illustrated in Fig. 4d.

2 $$\begin{aligned} \mathrm {Predicted\ Accommodation\ Depth\ }[D]= & {} a_{acc}((y_{P4}-y_{P3})-b_{acc}) \end{aligned}$$

3 $$\begin{aligned} \mathrm {Predicted\ Vergence\ Angle\ [deg] }= & {} a_{ver}(\sqrt{(x_{P4}-x_{P3})^2+(y_{P4}-y_{P3})^2}-b_{ver}) \end{aligned}$$

After determining these mathematical functions, we calculated the accommodation depth and vergence angle using experimentally found parameters and these functions. Considering the experimental data collected can be expressed as a time sequence, we analyzed the dynamic accommodation response and the change of vergence angle for different subjects as functions of time. In Fig. 4b,e, it is evident how well accommodation and vergence are predicted with mathematical functions over time for the same subject in Fig. 4a. The same procedure is followed for 9 subjects, and average values of accommodation and vergence were calculated for all target accommodation depths and vergence values. The average values of predicted accommodation depth/vergence angle and corresponding target accommodation depth /vergence angle are illustrated in Fig. 4c,f. As shown in Fig. 4c,f, the accommodation depth is predicted by the feature of the vertical distance between P3 and P4, with an average root-mean-square-error (RMSE) of 0.21 *D*.

### Accommodation and vergence prediction utilizing machine learning

**Figure 5:**
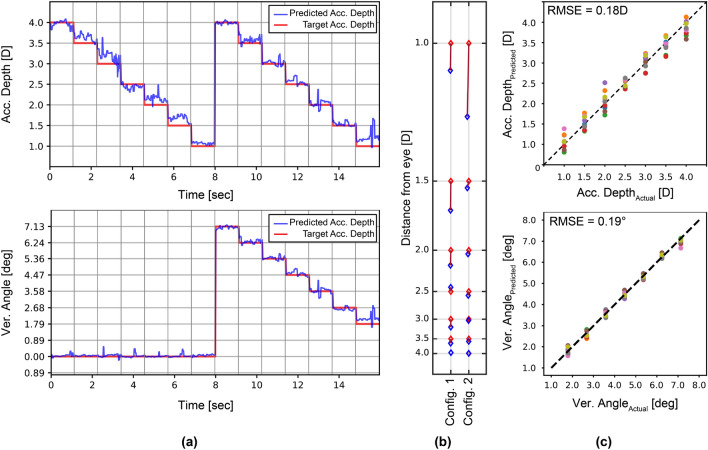
Accommodation and vergence predictions for 9 subjects using machine learning trained only with the subject’s own data (**a**) Our MLP model, trained on the subject’s own data, predicts the accommodation depth (top) and vergence angle (bottom) for the subject as a time sequence. (**b**) The error between the target and estimated points is illustrated using the average values of the predicted accommodation depth and vergence angle for this subject. (**c**) Average values of predicted accommodation depth and vergence angle are shown using our MLP model, trained on subjects’ own data, for all 9 subjects.

The accommodation and vergence can be related to specific features obtained from the locations of Purkinje images, as can be seen in Fig. 4. Even though vergence is predicted with low error, the accommodation depth error is high with this method. In the next step, we used ML regression algorithms where the inputs are the combination of 10 parameters (Purkinje reflection and pupil coordinates, and pupil shape) as defined above, and the outputs are the accommodation depth and the vergence angle. Different ML regression algorithms were tested for this purpose, and the multi-layer perceptron (MLP) was chosen as the best option. In the first method, the MLP algorithm was tested on the left eyes of all 9 subjects individually, and 30% of the each subject's data was used for training while the entire dataset obtained from the same user was used for testing. After splitting the data into training and testing, the batch size, number of epochs, number of hidden units at each layer, and the number of layers were specified by hyperparameter tuning. The predicted results from our MLP regression, using the locations of Purkinje reflections $(x_{P1}, y_{P1}, x_{P3}, y_{P3}, x_{P4}, y_{P4})$ as inputs, are expressed as a time sequence for one of the subjects in Fig. 5a. Figure 5b illustrates the average values of the predicted accommodation depth and vergence angle for 2 different configurations and how they deviate from the target accommodation depth and vergence angle. The same procedure was followed for all subjects. Figure 5c illustrates the average values of predicted accommodation depth/vergence angle and corresponding target accommodation depth (top) and vergence angle (bottom) by MLP regression for all subjects. The effect of pupil parameters on vergence and accommodation predictions was also investigated. Our aforementioned MLP model was used for analysis, but this time pupil parameters $(x_{PC}, y_{PC}, r_{1}, r_{2})$ besides the locations of the Purkinje images $(x_{P1}, y_{P1}, x_{P3}, y_{P3}, x_{P4}, y_{P4})$ were given as input to our MLP model. The results are included in Table 1 and Supplementary Fig. S1.

**Table 1:** Comparison of different methods and features on our dataset: (i) analytical function, (ii) Purkinje image locations as inputs to ML model trained only with the subject’s own data, (iii) Purkinje image locations as well as pupil parameters as inputs to the ML model trained only with the subject’s own data, (iv) Purkinje image locations as inputs to the ML model trained by excluding the subject’s own data, and (v) Purkinje image locations as well as pupil parameters as inputs to the ML model trained by excluding the subject’s own data.

	Depth error [*D*]	Angle error [deg]
Analytical function (Fig. 4)	0.21	0.19
ML trained with own data using P1, P3, P4 (Fig. 5)	0.18	0.16
ML trained with own data using P1, P3, P4, PC, PS	0.17	0.18
ML trained with others’ data using P1, P3, P4 (Fig. 6)	0.32	0.32
ML trained with others’ data using P1, P3, P4, PC, PS	0.36	0.4

**Figure 6:**
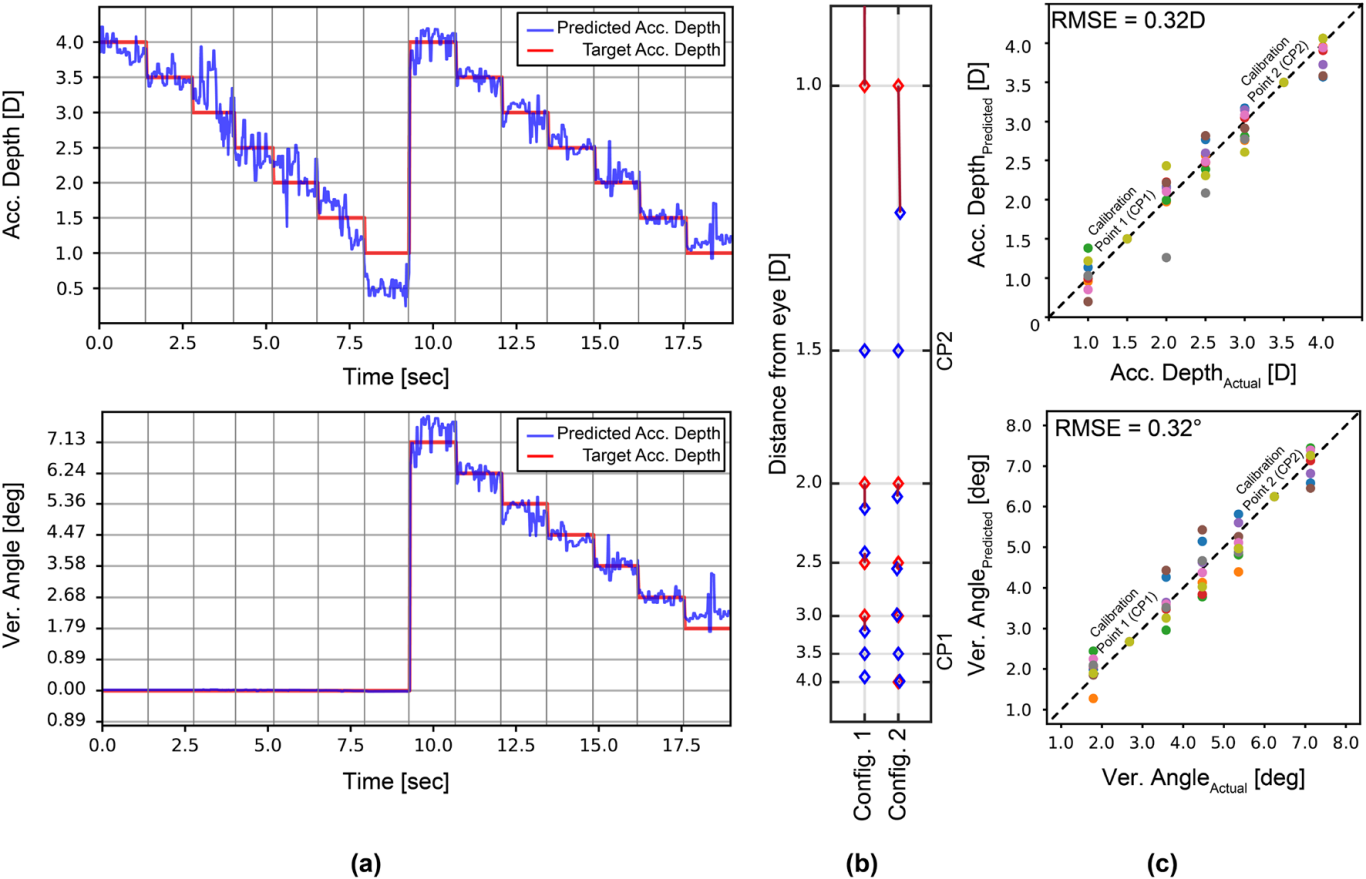
Accommodation and vergence predictions for 9 subjects using machine learning trained by excluding the subject’s own data. (**a**) Our MLP model, trained on data from other subjects and calibrated, predicts the accommodation depth (top) and vergence angle (bottom) for a single subject as a time sequence. (**b**) The error between the target and estimated points is illustrated using average values of the predicted accommodation depth and vergence angle for the same subject. (**c**) Average values of predicted accommodation depth and vergence angle using our MLP model, trained on data from other subjects and calibrated for all 9 subjects.

The MLP regression model was trained and tested for each subject individually using the first method. A more robust and generalized approach is to use different subjects’ data for training and testing. For this purpose, we applied leave-one-subject-out cross-validation (LOSO-CV) by training the model using data from 8 subjects and leaving the remaining subject out at each fold. Grid search was used to tune hyperparameters for the training set at each time, and the best hyperparameters were chosen. After training the model using the data from 8 subjects, the user was asked to look at the 2 target calibration points at 3.5 *D* and 1.5 *D*, selected to provide a large range while allowing subjects with myopia and hypermetropia to focus. The 10 parameters stated in the previous section were calculated by our image processing algorithm using the set of frames obtained for the 2 calibration points (CP). The accommodation depth/vergence angle values at the calibration points $(A_{CP1(pred)}, A_{CP2(pred)}, V_{CP1(pred)}, V_{CP2(pred)})$ are predicted by the ML model. Subsequently, calibration data were used to introduce a variable offset to the initial predictions by the ML algorithm to obtain the final predicted accommodation and vergence values ($A_{pred}$ and $V_{pred}$) using Eqs. (4) and (5):

4 $$\begin{aligned} \frac{A_{CP1(target)} - A_{calib}}{A_{CP1(pred)} - A_{pred}}= & {} \frac{A_{calib} - A_{CP2(target)}}{A_{pred} - A_{CP2(pred)}} \end{aligned}$$

5 $$\begin{aligned} \frac{V_{CP1(target)} - V_{calib}}{V_{CP1(pred)} - V_{pred}}= & {} \frac{V_{calib} - V_{CP2(target)}}{V_{pred} - V_{CP2(pred)}} \end{aligned}$$

where $A_{CP1(target)}$, $V_{CP1(target)}$, $A_{CP2(target)}$ and $V_{CP2(target)}$ are target accommodation depths and vergence angles at the calibration points, $A_{pred}$ and $V_{pred}$ are the predicted accommodation depth and vergence angle found by the regression model, and $A_{calib}$ and $V_{calib}$ are the accommodation depth and vergence angle after calibration. The results, where only Purkinje image locations were given as input to the MLP model, are analyzed in Fig. 6. The predicted results from our MLP regression are expressed as a time sequence for one subject in Fig. 6a. Figure 6b shows the average values of predicted and calibrated accommodation depth and vergence angle for 2 different configurations, and how they deviate from the target accommodation depth and angle. The same procedure is followed for all subjects. Figure 6c illustrates the average values of predicted accommodation depth/vergence angle and corresponding target accommodation depth (top) and vergence angle (bottom) by MLP regression for all subjects. According to the results, accommodation depth is found with an RMSE of 0.32 *D* and vergence with 0.32$^{\circ }$ on average.

Table 1 provides an overview of the vergence and accommodation estimation results using different methods and parameters. Detailed statistical analyses for different subjects are presented in Supplementary Fig. S1. It shows the RMSE error for different subjects using the 14 target test points as input to different methods. The first ML method (ML trained with the subject's own data using P1, P3, and P4) results in the lowest maximum RMSE error of 0.22 *D*. On the other hand, the second ML method using P1, P3, and P4, which uses a pre-trained model without the subject’s data, has a maximum error < 0.40 D. Note that the second method using P1, P3, and P4 performed better than the second method using P1, P3, P4, PC, and PS. For 3D display applications, in order to minimize the vergence and accommodation conflict to < 0.25 *D*, the accuracy of the method needs to be improved further, which can be done by enhancing the model using additional training data.

To investigate the impact of potential variability on the measurements, two of the subjects underwent additional tests. Their right eyes were also tested to determine whether eye selection (right or left eye) affects measurements. We noticed no meaningful difference between the predictions for the left and right eyes. The results of the first ML method for the right and left eyes of one of the subjects are compared with each other, as illustrated in Supplementary Fig. S3. Similar patterns can be observed in this figure across the 2 measurements. A randomized procedure (LEDs are turned on and off randomly) on the left eyes of 2 subjects is performed to avoid habituation to repeated stimulation. As expected, accommodation response times are longer in the randomized procedure. However, the predicted accommodation depth accuracy remained similar, as seen in Supplementary Fig. S2. System repeatability was tested by replicating experiments for the subjects and calculating the Pearson correlation coefficient. A fixed seed number was employed during calculations, guaranteeing the initial weights would not change. When we used the first ML algorithm, trained with the subject’s own data and took P1, P3, and P4 as inputs, the measurements from the same subject were highly correlated (r = 0.99, *p* < 0.001 for both subjects). Additionally, measurements from the second algorithm, using others’ data and taking P1, P3, and P4 as inputs, were still highly correlated for 2 subjects (r = 0.91, *p* < 0.001; r = 0.85, *p* < 0.001).

To quantify the effects of the Purkinje image locations on the accommodation and vergence estimations, we used a feature importance technique on the whole dataset (experimental results from 9 subjects) named permutation importance. This technique randomly shuffles the values of a single feature in the dataset, then uses the algorithm to make estimations from the permuted dataset and measures how much the model’s performance has decreased.

The relative weights of $x_{P1}$, $y_{P1}$, $x_{P3}$, $y_{P3}$, $x_{P4}$, and $y_{P4}$ are found as $0.23 \pm 0.005$, $0.13 \pm 0.004$, $1.0 \pm 0.035$, $0.50 \pm 0.017$, $0.69 \pm 0.020$, and $0.74 \pm 0.015$, respectively, in the first configuration using the permutation importance technique. Results reveal that the location of P1 is the least important feature for the accommodation estimation in the first configuration, as expected, given that P1 is the reflection from the cornea and does not include information about the curvature of the lens. On the other hand, it is important to find relative weights in the second configuration to see the vergence effect. The relative weights of $x_{P1}$, $y_{P1}$, $x_{P3}$, $y_{P3}$ , $x_{P4}$, and $y_{P4}$ are found as $0.47 \pm 0.015$, $0.06 \pm 0.002$, $0.18 \pm 0.005$, $0.09 \pm 0.005$,$1.0 \pm 0.019$, and $0.01 \pm 0.001$, respectively, in this configuration. The most important parameter is $x_{P3}$ for accommodation estimation, while $x_{P4}$ is an indicator for vergence estimation. It should be noted that these values are specific to the camera, LED, and eye positions that have been optimized for our application. Variations may occur with different configurations.

## Discussion

We proposed a simple setup for data collection and a real-time dynamic accommodation and vergence measurement system based on Purkinje reflections. Previous studies have explored the use of the first reflection (P1) from the cornea along with the third reflection (P3) from the anterior surface of the human crystalline lens^29,30^ or the fourth reflection (P4) from the posterior surface of the human crystalline lens for eye tracking systems^27,28,31^. However, these studies reported limited accuracy even when the system was calibrated for a specific user. Some of these studies^29,30^ mention the use of two-dimensional pupil size for vergence measurement, noting that vergence can be estimated by using changes in two-dimensional pupil size only. However, such methods are sensitive to ambient illumination changes and require calibration for each user.

In this study, we built a controllable RGB light source array-based setup for data collection to train our ML algorithm. We found that both P3 and P4 are important for accurate estimations, and the pupil center location is helpful for accurate vergence estimation. The proposed Purkinje reflection measurement setup is simple and uses only a camera and an NIR LED. We performed ZEMAX simulations to match our experimental results with the simulations, determining the optimum camera-LED-eye configuration for capturing all Purkinje images at accommodation depths covering 4 *D* to 1 *D* and vergence angles from 0 to 7 degrees.

In the first ML method, the data acquired from each subject was used in the training dataset for the ML algorithm. We demonstrate that an average RMSE of 0.17 *D* and 0.18 $^{\circ }$ is achievable for accommodation and vergence predictions when the subject’s pupils are monitored in subsequent trials. This method is an effective way of finding the accommodation depth and vergence angle, as only a certain portion of the dataset is used for the analysis, while all of the images in the dataset are used when the results are found using the analytical function.

In the second ML method, we performed LOSO-CV to determine the possibility of predicting one subject's accommodation and vergence with the information obtained from all other subjects, eliminating the need for user-dependent model training. Using the LOSO-CV method, only with two-point calibration data, the accommodation and vergence angle for a new user were predicted with an average accuracy of 0.32 *D* and 0.32 $^{\circ }$, respectively. It should be noted that due to the effects of the different orientations of the eye during the experiments and the intra-subject variability of human eyes, we have applied two-point calibration for both configurations to eliminate these effects. Nevertheless, subjects with refractive errors can affect the results as they cannot accommodate all target points. We tested our system on both eyes and observed similar patterns at various times using the subject’s own data. However, the camera-LED-eye configuration may vary between the right and left eyes, potentially affecting the system's performance. We made repeatability and reproducibility tests using the data from the same subject at different times. Even if the results indicate a high correlation between measurements, subjects’ alertness and misplacement of the eye may cause the measurements to deviate from each other.

Accommodation prediction of < 0.25 *D* can be considered adequate for AR/VR display applications since VAC remains within the acceptable limits^20^. The vergence prediction can be improved further by using a binocular system, such as those used in AR/VR headsets.

In conclusion, our prototype offers a reliable and accurate method for measuring dynamic accommodation and vergence based on Purkinje reflections. Our findings demonstrate the importance of using both P3 and P4 for precise estimation and provide a simple and efficient approach for implementing this technique. Our proposed method can be utilized for AR/VR displays and as a tool for vision research.

## Methods

**Figure 7:**
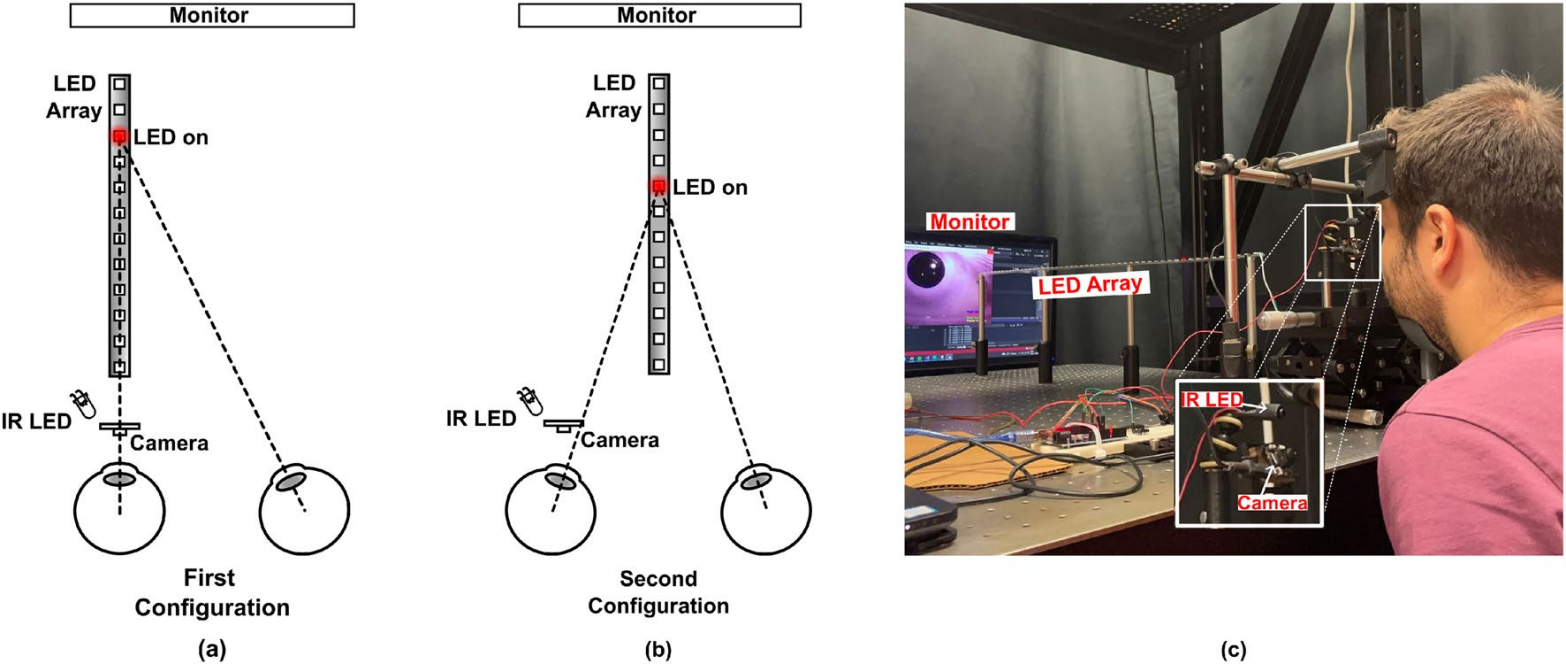
Schematic of the setup for measuring (**a**) accommodation effect, (**b**) accommodation and vergence combined effects, and (**c**) picture of the experimental setup.

In this study, we developed a setup to measure both accommodation and vergence using Purkinje images. One component of the setup is a controllable RGB light source array (WS2812B, Worldsemi Co.,Limited) placed in the visual field of the eye, covering target points from 4 to 1 *D* with 0.5 *D* intervals. The individual target LEDs on the RGB light source array were turned on and off, with each LED remaining on for 2 s. Participants were asked to accommodate their eyes to the LED turned on during the experiment. It was ensured that the LED brightness was adjusted to a level where the subject can accommodate without any disruption. Red LEDs at 620 nm were chosen as target points since the reaction time to red stimuli is short, and accommodation does not change significantly across different wavelengths^34^. One NIR LED source (TSAL6200, Vishay Intertechnology, Inc.) and one NIR camera (SQ11 Mini DV Camera, Dilwe1) were used to capture eye images from the participants. The peak wavelength of the NIR LED illuminating the eye is selected as 940 nm. It is eye-safe, compatible with silicon detectors, and invisible to the eye, making it a practical choice for our system. Purkinje reflections in the model and the experiments are matched by changing the dispersion of the ocular media used in the model. We recorded the images captured from the camera at 50 Hz for 2 s, corresponding to 100 frames for each data point for 9 subjects. Reaction time, the time it takes for the visual system to respond to a visual stimulus, was considered during experiments, and the frames captured before eyes react were discarded. Purkinje image locations were detected in an average of 15 ms. It took 16.4 s to train and test the data taken from one subject when the analysis was done individually. However, since the training with the other subjects’ data can be done before testing, it takes 0.4 s to predict the accommodation/vergence with the LOSO-CV method. All analyses were performed with Python version 3.11.2, except for the correlation analyses between the different extracted features and the accommodation/vergence performed with the “corr” function in MATLAB's Statistics and Machine Learning Toolbox (Math Works). Ray tracing simulations were carried out with ZEMAX OpticStudio version 19.4 SP1. All calculations were done with an Intel(R) Core(TM) i7-6600U CPU @ 2.60GHz located inside DELL E5470. The speed can increase if more efficient processing units are used.

The precise position of the controllable RGB light source array was altered for different applications. Firstly, we placed the RGB light source array almost on the nose-to-chin axis to observe the effects of both vergence and accommodation. In this configuration, eye rotation was present to fixate on a point and accommodate. Both effects were present in this configuration. Secondly, the RGB light source array was also positioned along the left or right eye to enable observation of the effect of accommodation on the locations of Purkinje images. This allowed us to observe monocular accommodation directly, which is useful for many different applications. In this configuration, we ensured that all light sources were clearly visible to the eyes and that no occlusion was present. It is important to note that Purkinje images are sensitive to head movements and different orientations. Therefore, correct head placement is necessary to prevent inaccurate results. To this end, we placed a monitor before capturing any images and asked the user to look at the specific part on the monitor and adjust their head location until their pupils were in the desired region on the monitor. We made adjustments using piezo stages when the user could not adjust their head correctly and minimized the errors caused by environmental effects and subject-specific variations. Our experimental setup schematics, designed for measuring the effects of both vergence and accommodation, as well as only the effect of accommodation are illustrated in Fig. 7, along with a picture of the setup.

9 healthy adults participated in this study with no ophthalmological diseases, except for myopia or hypermetropia. An oral disclosure and paper-based consent form were given to all participants prior to the study. Each participant gave written informed consent for participation in the study. All experimental protocols were approved by the Koç University Ethics Committee on Human Research and conducted according to the institutional guidelines. This study was conducted according to the principles of the Declaration of Helsinki.

### Supplementary Information

Supplementary Information.

## Data Availability

Data underlying the results presented in this paper are not publicly available at this time but may be obtained from the authors upon reasonable request. Requests for data and materials should be adressed to H.U.
