# Supplementary material for: Dynamic accommodation measurement using Purkinje reflections and machine learning
Source: Sci Rep. 2023 Dec 7;13:21625. doi: 10.1038/s41598-023-47572-0 (PMC10703819; doi:10.1038/s41598-023-47572-0)

Supplementary Table S1. Model Eye Parameters. Accommodation is denoted as A (in Diopters). Model parameters are consistent with publication[1]. Refractive index is modified for the NIR LED used in our experiments.

**Parameters**		**Value**
**Radius of curvature (mm)**		
	Anterior surface of cornea	7.72
	Posterior surface of cornea	6.5
	Anterior surface of lens	$10.2 - 1.75 \cdot \ln(A + 1)$
	Posterior surface lens	$-6 + 0.2294 \cdot \ln(A + 1)$
**Surface Thickness (mm)**		
	Cornea	0.55
	Aqueous	$3.05 - 0.05 \cdot \ln(A + 1)$
	Lens	$4 + 0.1 \cdot \ln(A + 1)$
	Vitreous	$16.4 - 0.05 \cdot \ln(A + 1)$
**Asphericity**		
	Anterior surface of cornea	-0.26
	Posterior surface of cornea	0
	Anterior surface lens	$-3.1316 - 0.34 \cdot \ln(A + 1)$
	Posterior surface lens	$-1 - 0.125 \cdot \ln(A + 1)$
**Refractive index (at 940 nm)**		
	Cornea	1.368
	Aqueous	1.3293
	Lens	$1.41157 + 8.0064 \cdot 10^{-5} \cdot (10 \cdot A + A^2)$
	Vitreous	1.329

Supplementary Figure S1. RMSE Error in Diopters based on 14 predictions for each subject using different algorithms.

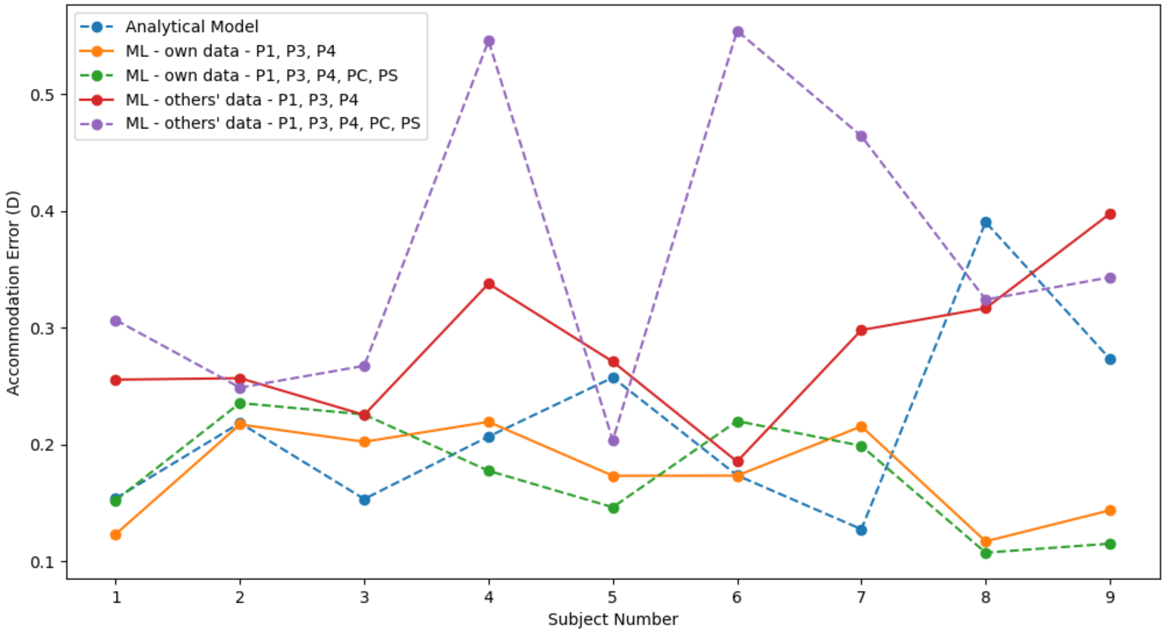

Supplementary Figure S2. Randomized procedure. Predictions for accommodation (top row) and vergence (bottom row). Target points are selected in random order. Input parameters are (P1, P3, P4) for (a) and (c), (P1, P3, P4, PC, PS) for (b) and (d).

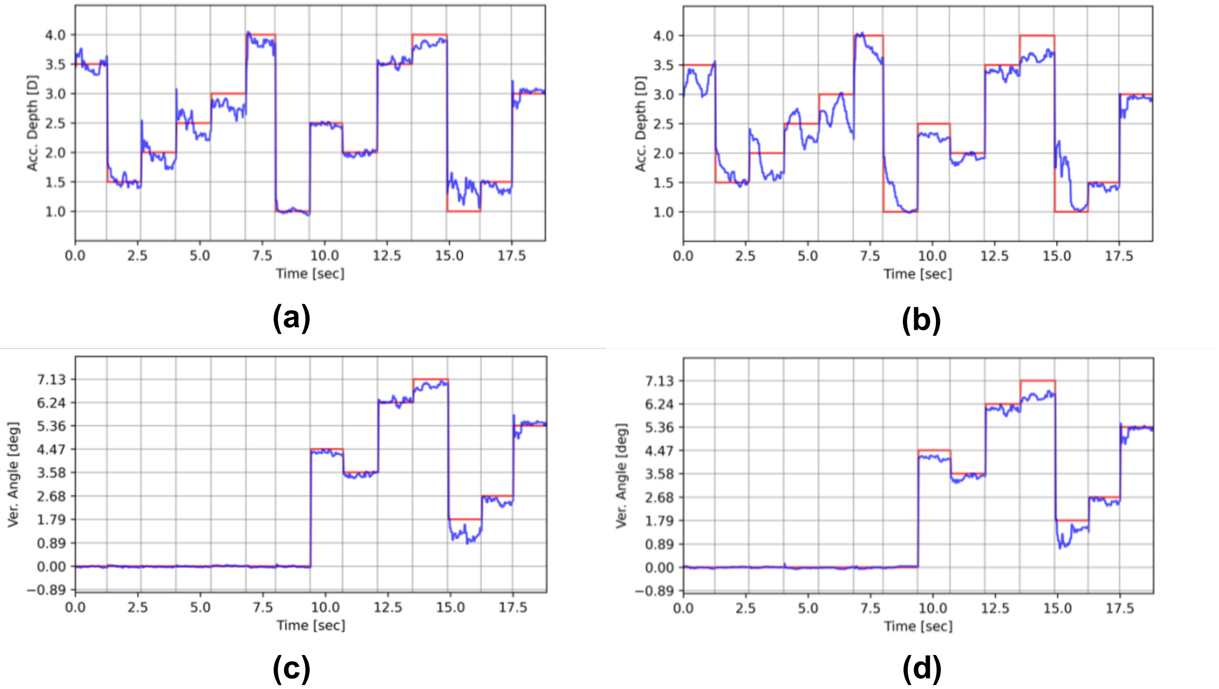

Supplementary Figure S3. Accommodation and vergence predictions for the right and left eye. Predictions for the left eye (a, c) and the right eye (b, d). Input parameters are (P1, P3, P4).

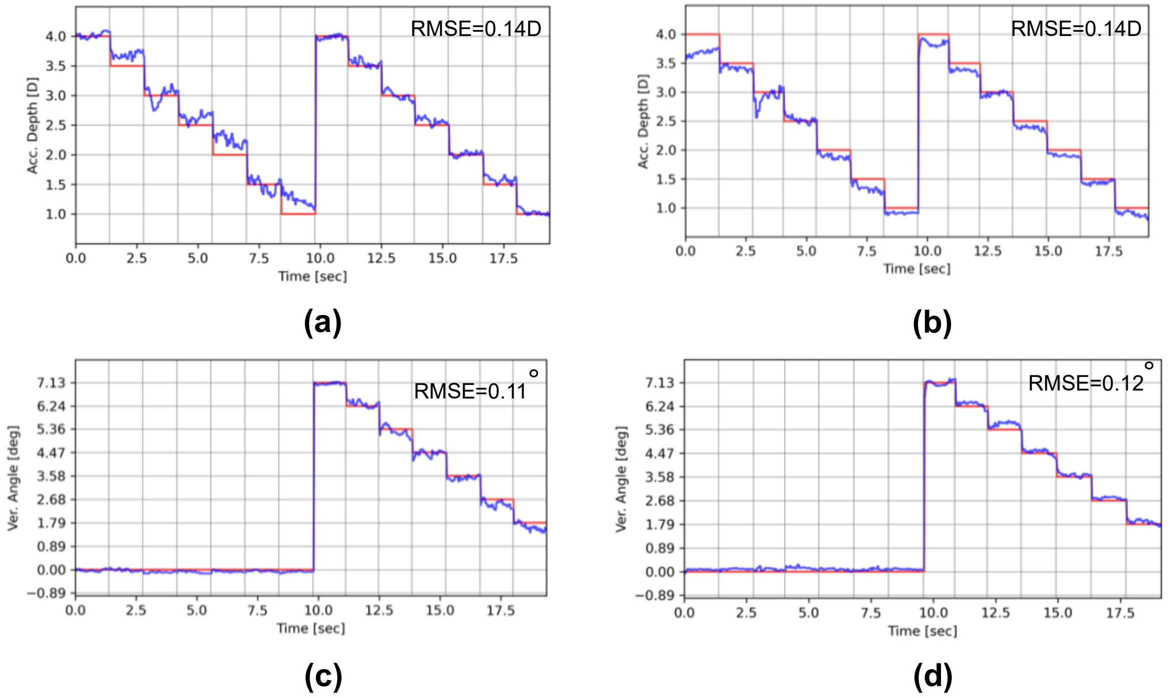

Supplement: Supplementary file 1 — Supplementary Information. [file 41598_2023_47572_MOESM1_ESM.pdf]
